# Clinical description and management of two terbinafine-resistant cases of tinea caused by *Trichophyton indotineae* from Finland

**DOI:** 10.1093/skinhd/vzag014

**Published:** 2026-03-31

**Authors:** Niina Kantola, Outi Varpuluoma, Elina Aho-Laukkanen, Suvi-Päivikki Sinikumpu, Laura Huilaja

**Affiliations:** Department of Dermatology, University Hospital of Oulu, Oulu, Finland; Department of Dermatology, University Hospital of Oulu, Oulu, Finland; Research Unit of Clinical Medicine and Medical Research Center, University of Oulu, Oulu, Finland; Nordlab Laboratory and Research Unit of Biomedicine and Internal Medicine, University of Oulu, Oulu, Finland; Department of Dermatology, University Hospital of Oulu, Oulu, Finland; Research Unit of Clinical Medicine and Medical Research Center, University of Oulu, Oulu, Finland; Department of Dermatology, University Hospital of Oulu, Oulu, Finland; Research Unit of Clinical Medicine and Medical Research Center, University of Oulu, Oulu, Finland

## Abstract

The first reports of terbinafine-resistant *Trichophyton indotineae* emerged from India in late 2017. Since then, superficial fungal infections caused by terbinafine-resistant *T. indotineae* have been increasingly reported in European countries as well. Some cases originate from travelling abroad, but local transmission also occurs. Currently, there are no internationally accepted guidelines for dealing with these cases. In some reports, itraconazole has been suggested as a drug of choice to treat these cases. Here, we report two cases in which griseofulvin was used successfully.

## Teaser

Fungal infections of the skin are common in people of all ages, and terbinafine has been commonly used to treat these cases. Recently, more cases with superficial tinea caused by *Trichophyton indotineae* resistant to terbinafine have been reported in Europe. We report two cases with clinical resistance to terbinafine in which griseofulvin with or without itraconazole was used successfully.

## Case report

Dermatomycoses are very common worldwide.^1^ The first reports of terbinafine-resistant *Trichophyton indotineae* emerged from India in late 2017, followed by similar reports from Asia, Europe and North America.^2,3^ Recently, cases were also reported from Sweden.^4^  *Trichophyton indotineae* has a high capability to mutate in the squalene epoxidase (*SQLE*) gene, leading to terbinafine resistance.^5^ Expert opinion recommends treating these cases with oral itraconazole,^3,5^ but some strains have shown variable susceptibility to azoles.^6^ Currently, mycological data of only 11 cases of terbinafine-resistant *T. indotineae* have been reported from Finland.^7^ We report two clinical cases from Northern Finland.

### Patient 1

A 50-year-old White man received amlodipine and losartan treatment for hypertension. Over the past year, a rash had appeared on various parts of his body. Due to diagnostic difficulties, a punch biopsy was taken in primary care, revealing fungal infection. Oral terbinafine 250 mg daily and hydrocortisone–natamycin–neomycin sulfate cream were initiated, with no clear benefit from a 2-month course.

**Figure 1:**
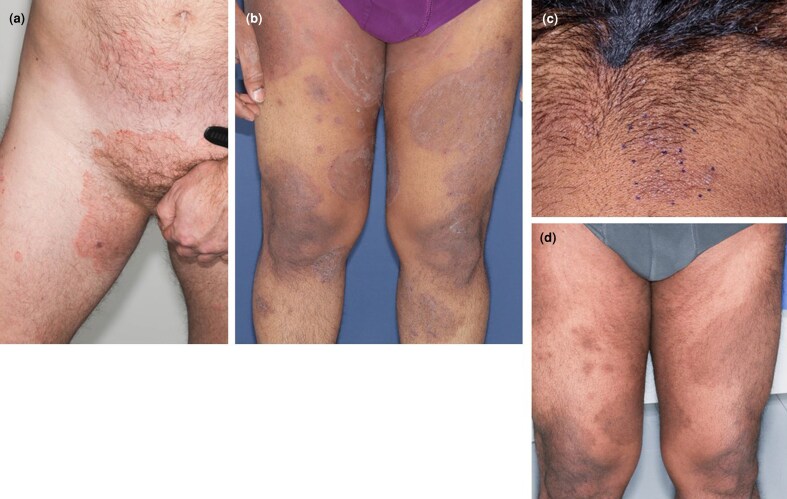
Clinical images of the lesions of terbinafine-resistant *Trichophyton indotineae* at the time of dermatological consultation. (a) Patient 1, (b) patient 2, (c) reinfection on the neck of patient 2 (dotted line) and (d) hyperpigmentation only in patient 2 after the treatment.

At a dermatology consultation in November 2023, widespread active tinea lesions were observed (Figure 1a). A nucleic acid amplification test (NAAT) was carried out using skin scraping samples from several sites (DermaGenius 2.0 Complete Multiplex kit; PathoNostics, Amsterdam, the Netherlands). The tests were positive for *Trichophyton interdigitale* and *Trichophyton mentagrophytes*. *Trichophyton indotineae* was also verified by sequencing the internal transcribed spacer (ITS) 1 and ITS2 regions of the strain isolated from fungal culture at the HUS Diagnostic Center (Helsinki, Finland). The method has been described in detail by Ala-Houhala *et al.*^8^

*SQLE* mutations suggestive of terbinafine resistance were not found (DermaGenius Resistance Multiplex kit; PathoNostics), but based on the patient’s history, the strain was considered clinically resistant to terbinafine. Oral itraconazole 100 mg daily for 4 weeks was initiated, in combination with ketoconazole shampoo washes, miconazole and hydrocortisone–natamycin–neomycin sulfate creams. The itching initially subsided but returned in conjunction with spreading tinea lesions. After a consultation with an infectious disease specialist, itraconazole was switched to oral fluconazole 150 mg daily. Due to poor response after a 1-month course, fluconazole was discontinued and oral ­griseofulvin 500 mg twice daily was introduced. After 5 months of ­griseofulvin treatment, the patient’s skin was completely healed, and the medications were stopped. At the 6-month follow-up he showed no signs of relapse.

### Patient 2

A 29-year-old otherwise healthy man originally from Sri Lanka with no history of skin diseases had developed an itching, ring-shaped rash cultured positive for *T. indotineae*. Prior to his referral to the dermatology department in April 2024, he had received a course of hydrocortisone–natamycin–neomycin sulfate cream, followed by oral fluconazole 150 mg daily and miconazole cream for a month, and finally oral itraconazole 100 mg twice daily for a week and 100 mg daily for 6 weeks after that. None of these regimens had any effect on his symptoms.

At a dermatology consultation, extensive lesions in several sites of the body, including the face, were noticed (Figure 1b). NAAT, fungal culture and sequencing of skin scrapings corresponded to those in patient 1, but an *SQLE* mutation suggestive of terbinafine resistance was detected. Oral griseofulvin 500 mg twice daily was initiated. However, after a month, the tinea lesions were spreading, and new lesions appeared. Oral itraconazole 100 mg twice daily was combined with griseofulvin. After that, the tinea lesions gradually started to thin and lighten. The itching did not return.

After 8 months of treatment, new lesions appeared on the previously unaffected neck (Figure 1c). The NAAT corresponded with the original samples, and the recurrent use of a lanyard was noticed as a possible source of reinfection. Ketoconazole shampoo and ointment were added as topical treatments. After 11 months of treatment in total, there was hyperpigmentation in the affected areas (Figure 1d), but no erythema or scaling, and the treatment was discontinued. At the 3-month follow-up he showed no signs of relapse.

## Discussion

We report two cases of terbinafine-resistant *T. indotineae* from northern Finland: one with a detected *SQLE* mutation and another where no mutation was detected using a commercial kit. However, this kit only detects five different *SQLE* mutations (Leu393Phe, Phe397Leu, Leu393Ser, Phe397Ile, Phe397Val) suggestive of terbinafine resistance, and as *SQLE* sequencing was not performed we cannot exclude the possibility of other, less common mutations in case 1.

Previously, the majority of European terbinafine-resistant samples have been shown to originate from Asia or the Middle East or have been found in Europeans who have recently visited endemic areas.^1^ In the second case, the patient was most likely infected in his home country of Sri Lanka as he had visited there shortly before the onset of his symptoms. However, the tinea of patient 1 most likely originated from local transmission as he had not been travelling due to COVID-19 pandemic restrictions. Recently, the significance of local transmission has been highlighted,^9^ and undetected circulation of *T. indotineae* has recently been reported in Sweden, for example.^4^

Although migration and travel have allowed the spread of terbinafine-resistant strains to Europe,^1^ there are also other possible explanations for the increase in cases of *T. indotineae* in Europe. Increased consumption of over-the-counter antifungal drugs, incomplete treatment cycles, and combined steroid, antifungal and antibacterial creams have contributed to the development of resistance.^10^ Moreover, poor treatment tolerance and treatment satisfaction,^3^ variability in antifungal quality and host immune dysfunction can also contribute to treatment failure.^11^

Fungal sampling is essential for the proper treatment of dermatophytosis, and emerging resistant strains further enhance its importance.^9^ Despite being recommended,^12^ no samples for dermatophyte detection had been taken prior to the initial treatment of patient 2, which most likely markedly delayed proper diagnosis and led to the worsening of the symptoms. The importance of susceptibility testing and monitoring trends in drug susceptibility across countries has been raised recently.^5,9^ Currently, no antifungal susceptibility testing for dermatophytes is available in Finland.

Terbinafine has been the first-line antifungal treatment for tinea for years due to its high efficacy and few side effects, but its efficacy has declined with the emergence of *T. indotineae*.^5^ However, it has been noticed that a higher daily dose of terbinafine (250 mg twice daily) could offset the effect of *SQLE* mutations in many patients and contribute to a positive therapeutic response.^5^ Despite this, itraconazole has been reported as the most effective drug against *T. indotineae* in recent case reports. However, adjusting the daily dose and duration of treatment is still largely empirical.^5^ In our patients, oral itraconazole was not efficacious. Nevertheless, the short treatment duration (4 weeks) or its low dose (100 mg per day) may have led to the failure of itraconazole in patient 1. We can only speculate whether its effect would have been better with a higher dose and an even longer course.^5^

Fluconazole and griseofulvin have also been tested for the treatment of *T. indotineae* but their reported clinical efficacy has not been very good.^1^ In our patients, due to the poor initial response and fear of side effects, griseofulvin was chosen in patient 1. That positive experience then led to the use of griseofulvin in patient 2. We can only speculate on the possible explanations for the good response to griseofulvin in our patients; it may originate from strain variability, host immunity or potential synergistic effects with itraconazole (in patient 2).

Itraconazole treatment (100 mg daily) for *T. indotineae* has required a particularly long treatment period, at least 6–8 weeks, to achieve total cure in most patients.^5^ Switching between drugs before that time may not be reasonable,^5^ but as demonstrated by our patients, it is not easy to refrain from treatment changes in clinical practice if visible signs of healing cannot be seen quickly. Currently, the combination of topical and oral antifungals is recommended as they are known to work synergistically,^13^ which was true for our patients as well. Nevertheless, further comparative studies are needed to set optimal treatment guidelines for *T. indotineae*.

As more terbinafine-resistant cases of tinea with local transmission are reported, it is crucial for clinicians to be aware of this possibility. Special attention should be paid to cases in which *T. indotineae* is found on the trunk or groin as those were shown to be the most common anatomical sites for recalcitrant tinea in a large international survey.^9^
